# Mental health of adolescents who abuse psychoactive substances in Enugu, Nigeria - A cross-sectional study

**DOI:** 10.1186/1824-7288-36-53

**Published:** 2010-08-10

**Authors:** Wilson C Igwe, Ngozi C Ojinnaka

**Affiliations:** 1 MBBS, FWACP (Paed), Consultant Paediatrician Nnamdi Azikiwe University Teaching Hospital, Nnewi, Nigeria; 2 MBBS, FWACP (Paed) Consultant Paediatrician and Child Neurologist, University of Nigeria teaching Hospital Enugu, Nigeria

## Abstract

**Background:**

Association between psychiatric morbidity and substance abuse among adolescent has been reported. However prevalence and pattern of such dysfunctions are unknown in our environment.

**Aims:**

To determine the prevalence of psychosocial dysfunction and depressive symptoms among adolescents who abuse substance and also note the influence of socio-demographic factors and type of substance on the pattern of dysfuction.

**Method:**

A cross-sectional study was carried out among 900 adolescents selected from 29 secondary schools in Enugu metropolis. A multi-stage sampling procedure was used to select the students. The student drug use questionnaire was used to screen respondents for substance abuse. Those who were abusing substance and matched controls (non substance abusers) were assessed for psychiatric symptoms using the 35-item Paediatric Symptom Checklist (PSC) and the Zung Self-rating Depression Scale (SDS). Social classification was done using the parental educational attainment and occupation.

**Result:**

A total of 290 students were current substance abusers. The substances most commonly abused were alcohol (31.6%), cola nitida (kola nut) (20.7%) and coffee (15.7%). Using the PSC scale, 70 (24.1%) subjects compared to 29 (10.7%) of the controls had scores in the morbidity range of ≥ 28 for psychosocial dysfuction. This was statistically significant (χ^2 ^= 17.57 p = 0.001). Fifty-four subjects (18.6%) had scores in the morbidity range of ≥ 50 for depressive symptoms using the Zung SDS compared to 21 (7.7%) of controls. This was statistically significant (χ^2 ^= 14.43, p = 0.001). Prevalence of dysfunction was not significantly related to age in both subjects and controls (χ^2 ^= 4.62, p = 0.010, χ^2 ^= 4.8, p = 0.10 respectively). Also using both scales, there was no significant relationship between psychosocial dysfunction and gender or social class in both subjects and control. The prevalence of dysfuction using both scales was significantly higher in multiple abusers compared to single abusers. Subjects abusing alcohol scored more on both scales compared to those abusing other substances.

**Conclusion:**

Prevalence of psychosocial dysfunction is higher in adolescents abusing substance compare to controls. The prevalence of psychiatric morbidity was not related to the age, gender or social classes in the study population.We advocate periodic screening of our adolescents for drug abuse regular evaluation of such group for possible psychopathology.

## Background

Use of illicit substances by a significant number of young people has been on the increase worldwide [1-3]. Increased use over the past decades, tendency towards multiple use of substance and earlier age of onset has all been noted amongst these youths [4,5].

During adolescence there is a high level of emotional distress and turmoil, with 6-20% of general adolescent population suffering various types of mental health problems[6,7]. Abuse of alcohol, tobacco, cannabis, or other psychoactive substances has been reported to contribute to the increase in the incidence of some psychosocial problems [8,9].

Adolescents that abuse substances have higher prevalence of psychosocial problems compared with the general adolescent population [10]. In a review of childhood psychiatric disorders in Port Harcourt, South-south Nigeria over a 4-year period, Stanley et al [10] noted that 25% of 411 patients had a substance-related psychiatric disorder. Alcohol and cannabis were the substance most commonly implicated in these patients. Similarly about 20% of psychiatric patients seen over a 1-year period in the out-patient department of University of Nigerian Teaching Hospital, Enugu, were adolescents who were abusing one substance or the other [11]. Depression was the most common disorder among these patients. Psychiatric disorders vary depending on the type of substances abused and the degree of involvement in the abuse. These disorders may be due to chronic use, acute intoxication or as a consequence of withdrawal from their use [8].

Epidemiological studies have noted that much of the substance abuse among youths takes place in schools [12,13]. Since substance abuse especially in early adolescence interferes with normal adolescent development, studies of school populations are therefore important for an understanding of the factors and dynamics associated with substance abuse in the adolescent population. There is however paucity of reports on psychosocial disorders associated with substance abuse among the general adolescent population in Nigeria. Available epidemiological studies of substance abuse psychiatric-related disorders among adolescents in Nigeria are hospital-based surveys conducted in different parts of the country [10,11].

It is, therefore, essential to document the prevalence of psychosocial disorders among substance-abusing adolescents who may not present with overt psychiatric manifestations since such adolescents may not appreciate their problems and therefore will not present to the hospital. Such studies will provide useful information that will assist health professionals in designing effective preventive measures.

## Materials and methods

The study was conducted among senior secondary school students in Enugu, Nigeria. The senior secondary school students were chosen because they provide the most accessible population of adolescents who would have abused various substances long enough to affect their mental health. Ethical approval was obtained from Ethics committee, University of Nigeria Teaching Hospital, Enugu. Written permission was obtained from Enugu State Ministry of Education, principals of participating schools and parents of respondents. Participation was voluntary and informed verbal consent was obtained from the students.

A multistage random sampling method was used to select the schools of respondents from 29 secondary schools in Enugu metropolis. The schools were stratified into three groups based on the gender of the students into boys', girls' and mixed schools. Two schools were selected from each stratum via balloting. Having determined the sample size, [14] 900 respondents were targeted for the study. One hundred and fifty respondents were thus selected from each of the six schools through systematic sampling. A total of 900 students were finally selected from the participating schools. The selection ensured a fair representation of the classes and gender of the respondents.

Data on substance use were collected using a modified WHO student drug use questionnaire with age range of 10-19 years [15]. The questionnaire has been tested and found valid for epidemiological studies in Nigeria [16]. Substances included in the survey were alcohol, nicotine (in form of kola nut and coffee), inhalants, tranquillizers, cigarettes and cannabis. Current substance abusers (Those who used any of the substances in the past twelve months and were still using them within the 30 days preceding the survey) were documented. The mental health of these substance abusing respondents was compared with those of non-substance abusing adolescents using Pediatric Symptom Checklist (PSC) and Self-rating depression scale (SDS). The Pediatric Symptom Checklist (PSC) is a brief psychosocial screening questionnaire with 35 items that identifies children with psychosocial problems [17]. It does not classify children into specific diagnostic categories of psychiatric disorders. A total score of 28 or more was taken as an indication of significant psychosocial impairment. The Zung Self-rating Depression Scale (SDS) is a 20 items self-administered questionnaire developed to measure subjective symptoms of depression [18]. The scale has indices which range from 25-100. Values from 50 and above are taken as indication of depressive symptoms. Values in the range of 50-59, 60-69 and ≥70 and above are regarded as mild, moderate and severe depressive symptoms respectively. Both instruments have been standardized and widely used in various studies in Nigeria [19-21]. Social classification was derived from the parental educational attainment and occupation [22].

The data was collated in a master sheet and fed into a Statistical Package for Social Sciences-11 (SPSS-11) computer software for analysis. Tests of association using chi-square test were carried out between the prevalence data and psychosocial variables while t-test was used for comparison of means of subjects and controls. For all statistical analysis, p-values less than 0.05 were considered significant.

## Results

Response was obtained from 860 (95.6%) of the 900 respondents. Of these, 499 (57.4%) were males while 360 (42.6%) were females. Three hundred and sixteen (36.7%) had never used any of the substances in their lifetime while two hundred (29.5%) of the respondents were lifetime users (Lifetime use is defined as ever use of a substance in one's lifetime) [15].

Analysis of 290 (33.7%) current substance abusers who were screened for psychiatric morbidity using the scales was done. Their ages ranged from 11 to 19 years. The mean age of these students was 16.9 years (SD = 1.7, range 11-19) with 81, 176 and 33 students in the early, middle and late adolescent age groups respectively.There was no significant difference in mean age between subjects and controls (t = 18.5, p > 0.05). There were 188 (64.8%) males and 102 (35.2%) females giving a male: female ratio of 1.8:1. There was no significant difference in gender distribution between subjects and controls (χ^2 ^= 0.33, p = 0.50). The social class distribution shows that 103 (35.5%), 90 (31.0%), and 97 (33.5%) of the subjects belonged to the upper (I and II), middle (III) and low (IV and V) social classes respectively. There was no significant difference in social class distribution between subjects and controls (χ^2 ^= 0.01, p = 0.90).

Among the subjects, 75.2% were multiple substance abusers while 24.8% were single substance abusers. Alcohol recorded the highest single abuse prevalence of 31.6% while cannabis was the least (4.1%) (Table 1).

**Table 1:** Substance abuse prevalence according to type of substance

Substance	Current use (%)
Alcohol	272 (31.6)
Cola nitida (Kola nut)	178 (20.70
Coffee	135 (15.7)
Cigarette	123 (14.3)
Inhalants	77 (9.0)
Tranquillizers	64 (7.4)
Cannabis	35(4.10

The range for PSC score was 2-37 with a mean of 16-68 ± 7.1 and 2-35 with a mean score of 16.23 ± 6.63 in both subject and control respectively. This however was not statistically significant with p > 0.05. On the SDS scale, range was 16-67 with a mean of 41.64 ± 9.0 and 30-75 with a mean of 48.71 ± 8.33 in both subject and control respectively. This was stistically significant (t = 4.02; p = 0.001).

Table 2 shows that 70 (24.1%) subjects had scores in the morbidity range of ≥ 28 for psychosocial dysfunction on the PSC screening scale compared to 29 (10.7%) of the controls. This was statistically significant (χ^2 ^= 17.57 p = 0.001). The highest prevalence of psychosocial dysfunction was recorded among the mid adolescent age group in both subjects (60%) and controls (51%). There was however no statistically significant difference between the proportions of students with psychosocial dysfunction and the age groups in both subjects and controls (subjects χ^2 ^= 1.203, df = 2, p = 0.30; controls χ^2 ^= 7.75, df = 2, p = 0.07).

**Table 2:** Mental health problems according to age group.

Age group (yrs)	Subjects		Controls	
	PSC n(%)	SDS n(%)	PSC n(%)	SDS n(%)
11-13	24(34.3)	19(35.2)	8(27.6)	6(28.6)
14-16	42(60.0)	33(61.1)	15(51.7)	9(42.9)
17-19	4(5.7)	2(3.7)	6(20.7)	6(28.6)

**Total**	**70**	**54**	**29**	**21**

Fifty-four (18.6%) of the subjects had scores in the morbidity range of ≥ 50 for depressive symptoms on the SDS compared to 21 (7.7%) controls. This was statistically significant (χ^2 ^= 14.43, p = 0.001). About 40% of the subjects scored in the moderate to severe range on the SDS (60-69 and ≥ 70) compared to 13% of controls. The mid adolescent age group (14-16 yr olds) had the highest prevalence rate for depressive symptoms in both subjects and controls. Prevalence of dysfunction was however not significantly related to age in both subjects and controls (subjects χ^2 ^= 4.62, df = 2, p = 0.10, controls χ^2 ^= 4.81, df = 2, p = 0.10).

Table 3 shows the distribution of subjects and controls with psychosocial disorders according to gender. Among the subjects, 44 (62.9%) males compared to 26 (37.1%) females had psychosocial dysfunction on the PSC. For the controls, 20 (69%) males compared to 9 (31%) females were affected. There was no significant relationship between psychosocial disorders and gender in both subjects (χ^2 ^= 0.517, df = 1, p = 0.50) and controls (χ^2 ^= 0.594, df = 1, p = 0.50). Thirty-three, (61.1%) males compared to 21 (38.9%) females among the subjects had depressive symptons on the SDS. Similarly, 12 (57.1%) males compared to 9 (42.9%) females were affected among the controls. There was no significant relationship between scores and gender in both subjects (χ^2 ^= 0.402, df = 1, p = 0.50) and controls (χ^2 ^= 3.081, df = 1 p = 0.10).

**Table 3:** Mental health according to gender.

Gender	Subjects		Controls	
	PSC n(%)	SDS n(%)	PSC n(%)	SDS n(%)
Male	44(62.9)	3361.1)	20(69.1)	12(57.1)
Female	26(37.1)	21(38.9)	9(31.0)	9(42.9)

**Total**	**70**	**54**	**29**	**21**

Table 4 shows the distribution of subjects and controls with psychiatric morbidity according to their social class. There was no significant association between social class and scores the subjects using both scales (PSC χ^2 ^= 0.896, df = 4, p = 0.95; SDS χ^2 ^= 1.850, df = 4, p = 0.80). Similarly, there was no relationship between social class and scores among the controls (PSC χ^2 ^= 0.896, df = 4, p = 0.95; SDS χ^2 ^= 1.610, df = 4, p = 0.80).

**Table 4:** Mental health and social class.

Age group (yrs)	Subjects		Controls	
	PSC n(%)	SDS n(%)	PSC n(%)	SDS n(%)
I.	12(17.1)	10 (17.5)	3(10.3)	2(9.5)
II.	13(17.3)	10(18.5)	5(17.20	4(19.1)
III.	23(31.2)	19(34.2)	11(37.9)	7(33.3)
IV.	15 (21.2)	10(18.4)	6(20.7)	5(23.8)
V.	3(13.2)	8(11.40	5(9.3)	4(13.8)

**Total**	**70**	**54**	**29**	**21**

Table 5 shows the mental health of the subjects according to substance abuse types. Among subjects that were dysfunctional on the PSC scale, 82.9% were multiple substance abusers compared to 17% in single substance abusers. The prevalence of dysfunction was significantly higher in multiple abusers compared to single abusers (χ^2 ^= 3.9, df = 1, p = 0.05). Among the single substance abusers with dysfunction, 50%, 25%, 8.3% and 16.7% were abusing alcohol, tranquillizers, kola nut and cigarette respectively. Similarly, about 85% of subjects that were dysfunctional on the SDS were multiple substance abusers compared to 14.8% in single substance abusers. The prevalence of dysfunction was significantly higher in multiple substance abusers compared to single substance abusers (χ^2 ^= 3.57, df = 1, p = 0.05). Among the single substance abusers with dysfunction, 37.5% were abusing alcohol while 25%, 12.5% and 25% were abusing tranquillizers, kola nut and cigarette, respectively.

**Table 5:** Mental health according to substance abuse types.

Substance abuse	PSC N (%)	SDS N (%)
Single	12(17.1)	8(14.8)
Multiple	58(82.9)	46(85.2)

**Total**	**70**	**54**

## Discussion

The present study noted a higher prevalence of psychiatric morbidity in substance abusers compared to non-abusers on both scales. Current substance abusers were used because this group of respondents was more likely to experience problems associated with substance abuse in the adolescent [10,23,24]. The findings that the rates of psychiatric disorders are much higher among adolescents with current substance abuse have been replicated in many studies [8,20,25-29].

Depressive symptoms were noted in about 18% of the substance abusers compared to 7.7% in non-substance abusers giving a ratio of 2.34:1. About 40% of the abusers scored in the moderate to severe range compared to 7(13%) in non substance abusers. Rey et al (29) reported that 32% of substance abusers compared to 12% of non-abusers (2.67:1) were depressed. Similarly, Wang et al (30) obtained a prevalence of 58.3% among abusers compared to 17.7% (3.3:1) in non abusers. Our study gave a lower ratio when compared with these reports.

Reports on the association of socio-demographic variable such as age, gender and social class with psychiatric morbidity among substance abusing adolescents have been inconsistent in several studies [27-32]. Goodman et al [27], noted increasing age and low socioeconomic status to be strongly associated with psychiatric morbidity. Boys et al [28] and Rey et al [29] in separate studies also noted age as a significant risk factor. Shrier et al [32] noted a significant association between depression and female gender while Ohaeri et al [33] reported an association with male gender and low social class. The present study did not observe any relationship between psychiatric morbidity and any of the socio-demographic variables.

Multiple substance abuse has been noted as one of the risk factors for psychiatric morbidity in substance abuse [28-30,33]. The findings of the present study agree with the above report. This may be due to the additive pharmacological toxicities of the substances, acute intoxication, or as a consequence of withdrawal from chronic use[8]. Boys et al [28] examined the co-morbidity of dependence on single and multiple substances with emotional disorders and found that the risk for psychiatric morbidity was greater among individuals who were uniquely abusing cigarette, alcohol or Cannabis. Goodman and Capitman [27] also reported that tobacco abuse is most pronounced among adolescents with emotional disorders. The present study did not observe any significant association between the abuse of alcohol, kola nut or cigarettes with any psychiatric morbidity. This may be due to the fact that the figures of prevalence for the individual substances were small, thus precluding meaningful analysis. It is, however important to note that those who abused alcohol, kola nut or cigarettes were the ones who were depressed among the single substance abusers.

In conclusion substance abusing adolescents seem to have a higher prevalence of mental health symptoms compared to their peers who do not use substances. Multiple drug users have higher prevalence of psychiatric morbidity compared to single substance abusers. Socio-demographic factors do not seem to influence the prevalence of psychiatric morbidity amongst substance abusing adolescents. There is therefore a need for school-based educational program aimed at promoting healthy lifestyles devoid of substance use. In settings without overt manifestations of psychiatric symptoms efforts should be made to evaluate substance abusing adolescents for a possible psychiatric morbidity. Early case identification, counselling or referral for treatment at specialized centers will help reduce incidence of overt psychiatric manifestations of substance abuse.

## Competing interests

The authors declare that they have no competing interests.

## Authors' contributions

Both authors contributed to the conception of the study. WCI led in the collection of the data and wrote up the initial draft of the manuscript while NCO supervised the write-up, reviewed the statistics and corrected the reviewed manuscript. Both authors read and approved the final manuscript.
